# Description and comparison of physical activity from self-reports and accelerometry among primary school children in Kilimanjaro, Tanzania: a pilot study

**DOI:** 10.12688/aasopenres.13118.4

**Published:** 2021-05-17

**Authors:** Mary Vincent Mosha, Elizabeth Kasagama, Philip Ayieko, Jim Todd, Sia E. Msuya, Heiner Grosskurth, Suzanne Filteau

**Affiliations:** 1 Institute of Public Health, Kilimanjaro Christian Medical University College, Moshi/ Kilimanjaro, +255, Tanzania; 2 Mwanza Intervention Trials Unit (MITU), London School of Hygiene & Tropical Medicine, Mwanza/ Tanzania, +255, Tanzania; 3 London School of Hygiene & Tropical Medicine, London, WC1E 7HT, UK

**Keywords:** Children, self-reports, accelerometer, physical activity, validation, Tanzania

## Abstract

**Background**: Self-reports are commonly used to assess physical activity in children. Existing self-reports for physical activity have not been validated for primary school children in Tanzania. To understand if primary school children can accurately report their physical activity, we examined the validity of self-reported physical activity against accelerometer measured physical activity.

**Methods**: A community-based cross-sectional study was conducted from May to July 2018. We conveniently selected four primary schools in Moshi municipal and Moshi rural districts in Kilimanjaro, Tanzania. From these districts, 51 children aged 9 – 11 years were randomly selected. A self-reported questionnaire was used to collect physical activity-related variables. Children wore accelerometers for seven consecutive days to capture physical activity movements. Spearman’s rank test and Bland Altman plots were used for assessing validity and agreement between self-reports and accelerometer moderate to vigorous physical activity (MVPA).

**Results**: The study participants' mean age was 10 (SD=0.8) years, and 32 (63%) were girls. A significant positive correlation was found between self-reports and accelerometer MVPA (rho=0.36, p=0.009). The mean total of weekday minutes in MVPA from accelerometers was higher than from self-reports, 408 (SD = 66) versus 261 (SD = 179).

**Conclusions**: This study found a significant positive correlation between self-reports and accelerometers. Self-reports are prone to errors due to recall bias, which interferes with their validity. More research is needed to develop better self-reported measures with specific activities that children can easily remember. Also, researchers should carefully consider the inherent limitations in the validity of self-reports.

## Introduction

Physical activity in children is the key to better health. Active children gain health benefits, including cardiorespiratory, muscular fitness and bone health. Physical activity plays a vital role in preventing non-communicable diseases (NCDs) and avoiding weight gain ^1^. The World Health Organization (WHO) recommends an average of at least 60 minutes per day of moderate to vigorous activities (MVPA) for 5–17-year-olds to benefit their later life ^2– 4^. Studies in low- and middle-income countries (LMICs) reported low physical activity levels and high sedentary behaviours in children ^5, 6^. In Tanzania, 82.1% of school-going children are not meeting the recommended physical activity levels, according to self-reports ^7^.

Physical activity is a complex behaviour that includes day-to-day variations of activities, and these activities may not be easily remembered by children ^8^. Tools for measuring physical activity include subjective measures such as self-reports, proxy reports by parents and teachers, activity diaries and recall. Self-reports are prone to errors due to significant day-to-day variations and inaccurate estimation of physical activity levels ^9, 10^. The selection of a suitable assessment questionnaire for physical activity and instruments is based on the target population under study, respondent burden, cost-effectiveness and type of information to be collected.

Over the years, accelerometers have been increasingly used in high-income countries to assess physical activity in children ^11, 12^. However, in LMICs, few studies have used objective measures to assess physical activity. Objective measures include motion sensors such as heart rate monitors, accelerometers and pedometers. Heart rate monitors are cheap to use, but they have shown a weak relationship with energy expenditure. Pedometers only capture steps taken to provide estimates of activity levels. Accelerometers are more valid than self-reports in estimating physical activity ^8, 9^. Finally, the doubly labelled water method is a gold standard to measure energy expenditure but is expensive and requires specialist techniques ^13^.

The validity of self-reports for assessing physical activity in primary school children has not been fully explored in Tanzania. It is unclear if children from Tanzania can accurately report their physical activity or estimate minutes spent in physical activity. Therefore, this study aimed to describe the physical activity, then compare and determine the validity of a self-reported questionnaire to measure physical activity in Tanzanian primary school children, using an accelerometer as the reference method.

## Methods

### Study design and setting

A community-based cross-sectional study was conducted in two purposely selected Kilimanjaro region districts in the Northern part of Tanzania, Moshi municipal and Moshi rural district. We conveniently selected two primary schools (one private and one government-funded) from each section.

### Study participants

School children were recruited through a simple random technique between May to July 2018. A sample of 80 children (20 from each school) aged 9–11 years was randomly selected from the school attendance registers. The children’s parents were contacted and provided a detailed explanation of the study aims and procedures. After that, the research team sent the children home with the information sheet and consent form for parents to sign, thereby acknowledging their child permission to participate in the study. Only children who were day-students (not boarding) were eligible for the study.

### Study variables

Our primary study variable was minutes per day spent in MVPA, obtained from self-reports and accelerometry.

### Data collection methods and tools

For this study, a questionnaire from the International Study on Childhood Obesity, Lifestyle and Environment (ISCOLE) was adapted and modified ^14^. We reviewed the ISCOLE physical activity questions to check for appropriateness in this cultural context and its applicability amongst primary school children in Tanzania. This questionnaire was used in several high-income countries and one African country (Kenya). The focus during modification was to retain those questions that were descriptive enough for children to understand and related to durations and participation in different activities. We made changes to account for relevant and regular exercise types and the structure of questions. These modifications involved rewording some questions, and removing questions that were not appropriate, e.g., one question asked, “How much time did you spend outside before school or before bedtime?” This question was removed because it did not necessarily imply physical activity. We also removed questions that were not directly related to the study aim, e.g., “I can ask my parent or other adults to do physically active things with me”, “I find exercise a pleasure activity”.

A draft modified questionnaire, available as *Extended data* ^15^, was shared with the Regional school health coordinator for review and advice and then piloted with 15 school children to check for comprehension and relevance. Children were asked to indicate activities during typical days in their lives, stratified by the school and non-school days. Inputs from the students helped modify the flow and how some questions were asked. The tool was then returned to the 15 students, and after adjustment, the pilot's final questionnaire was developed.

## Physical activity measurements

### Self-reports

The final questionnaire was designed to collect information on multiple physical activities, including types, frequency and duration. The last sets of questions in the modified questionnaire focused on activities in a typical week (weekdays/ school days and weekends). The questions were as follows: “how many days did you participate in physical education classes (PE),“ how long did it take to walk to school?”, “participation in after school activities (e.g. house chores)”, “when you are at school, during break time do you participate in any type of physical exercise such as playing netball, football, skipping etc. or any other activity?”, and “how many days were you physically active for 60 minutes a day?”, “how many days did you watch television?”, “for how long did you watch television?” “how many days did you play video games?”, “for how long did you play video games or use a computer for non-school activities?”

### Accelerometry

Children were instructed how to wear the triaxial accelerometers (ActiGraph, wGT3X-BT Pensacola, FL). An accelerometer is a device used to measure physical activity movement. Researchers gave teachers and parents instructions (verbal and written) to assist their children with the accelerometer attachment. Further, researchers contacted parents/teachers every morning to ensure they reminded their child to wear the accelerometers. Children attached the accelerometers with an elastic band on their right hip. Children were instructed to remove the accelerometers when bathing or engaging in any water activity such as swimming. Accelerometers were set to collect data from 06:00 AM to 09:00 PM (bedtime) except for the initiation day when accelerometers commenced collecting data from 09:00 AM. Children wore accelerometers for seven consecutive days. When returned, data from each accelerometer were uploaded to the computer using Actigraph software, ActiLife version 6.13.4.

### Statistical analysis

Self-reported activity data were entered into Excel, and accelerometer data were exported to Excel; both were then imported into STATA IC version 15.1 (StataCorp, College Station, TX, USA) for analysis.

Descriptive statistics were used to summarize the demographics and physical activity data from self-reports and accelerometers. The distribution of data was checked using the Shapiro Wilk test. It showed that daily average MVPA data from the accelerometer were normally distributed (p = 0.34). In contrast, for self-reports MVPA data were positively skewed, indicating high activity levels in some children (p < 0.05). Mean (standard deviation), and median (interquartile ranges) were used as appropriate. Different frequencies and proportions were used for categorical variables.

***Estimating physical activity MVPA***

*Self-reports MVPA*

Total time spent in different activities was calculated from the questionnaire responses. Total weekday MVPA was defined as the sum of minutes for walking to school for five school days (Monday – Friday) and reports of being physically active for at least 60 minutes each day of the week. The number of minutes used to walk to school was summarized from the categorical responses, and the midpoint was calculated. For example, a response of 15–30 minutes of walking to school was considered as 22.5 minutes. Being physically active for at least 60 minutes for each school (weekday) was calculated by multiplying the number of days and minutes. For example: if the child reported being active for three days, we multiplied this by 60 minutes to get 180 active minutes. The average minutes of MVPA were estimated by dividing the total time of MVPA by the five days of the week.

***Accelerometry MVPA***

*Data reduction and scoring*

The raw activity data were reduced into 15-s epochs data for analysis, then converted to “.agd” files and imported into “CSV” and Excel sheets using Actigraph software, ActiLife version 6.13.4. Evenson’s cut points for children used to categorize activities in counts per minute (CPM); 0–100 (Sedentary), 101–2295 (Light), 2296–4011 (Moderate) and >4012 (vigorous) ^16– 18^. After that, total time spent in moderate and vigorous physical activity (Total MVPA) was estimated. Spike tolerance was set to zero, and a minimum length of the non-wear period was set to 60 minutes of consecutive zeros to allow for interruptions. The minimum wear time was set to 10 hours, and children were included in the analysis if they had sufficient and valid accelerometer data with a minimum of three weekdays and at least one weekend day. For weekends children were included in the analysis if they had adequate accelerometer data with at least one weekend day. Children who did not meet these criteria were excluded from the analysis.

Using an Actigraph software and considering the Tanzanian primary school daily timetable, we applied filters to define time blocks of activities from the accelerometer output. The blocks were matched the self-reports questions to countercheck if reported activities were confirmed by accelerometer MVPA, e.g., walking to school.

**Figure 1.**
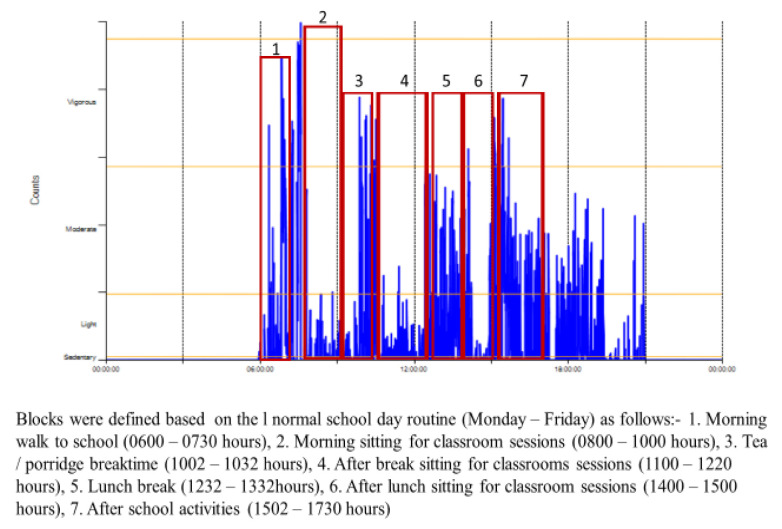
Illustrates the accelerometer output for a single child in one weekday (school day), with defined blocks of activities.

Figure 1 presents an example of how the accelerometer captured activities and how this varied by time blocks. The graph was constructed using data from one child for one day of the week. The period with no bars means the child was not active or the child did not wear the device. The horizontal axis shows the time in 24 hours, and the vertical axis shows the levels of activities achieved.

***Correlation between self-reports and accelerometer MVPA.*** To examine validity, we included only school days (weekdays), based on the assumption that children spend most of their time in schools and routinely participate in physical education classes. During weekend days, children are engaged in unstructured activities, which might be difficult for them to recall. We used scatter plots and Spearman’s rank test to check for the correlation between overall weekday physical activities (MVPA) from self-reports and those measured by the accelerometer. The strength of correlations were ranked as small > 0.1, moderate > 0.3 and strong > 0.5 ^19^. Also, Bland–Altman plots were used to assess the agreement between average weekday self-reported MVPA and accelerometer measured MVPA.

***Variability between self-reports and accelerometer blocks.*** Box-and-whisker plots were constructed to show the variability between self-reported responses (walking to school, exercise during tea break, exercise during lunch and after school activities) with the matching time block of activities from the accelerometer MVPA across the days of the week. Mann Whitney U test was applied to assess the significance of the difference in the median between children who reported participating in activities versus not participating.

***Mean weekday MVPA.*** The mean weekday MVPA (minutes per week) for both self-report and accelerometer data were calculated. Student’s T-test (for two groups) and ANOVA (for age only; > 2 groups) were used to compare the mean weekday MVPA from self-reports and accelerometers by sex, age, school location, school type, walking to school, exercise during breaks, after school activities and participation in physical education sessions. We accounted for the clustering effect of children within schools. We also performed post hoc pairwise comparison using the Bonferroni test for comparing the differences in mean total MVPA by age categories. There were no significant differences in means across age categories.

***Sub-group analysis***

*Associations between child-level variables and accelerometer MVPA*

We performed further analysis to explore the associations between weekday accelerometer MVPA and different child-level variables (sex, age, school type, school location and walking to school). Univariable and multivariable linear regression models were performed. We accounted for the repeated accelerometer measures for the same child on different days of the week. A child was regarded as a cluster since the repeated accelerometer measurement were nested within a single child. Regression coefficients from the linear regression, 95% confidence intervals (95% CI) and intra-class correlations were presented.

### Ethics approval and consent to participate

All ethical procedures concerning human participants were followed. Ethics approval was obtained from the National Institute for Medical Research (NIMR), Tanzania certificate number: IX/2735 on 27/03/2018 and the Kilimanjaro Christian Medical University College Ethics Committee (KCMUCO) certificate number: 2225 on 21/09/2017. School permission was obtained from the regional medical officer, district education officers and school authorities. All parents of participating children signed written informed consent for their children to participate. Children were asked to sign a brief written assent to participate in the study.

## Results

### Demographics and child characteristics

A total of 51 primary school children were enrolled in the study, interviewed and wore accelerometers. Of the 80 parents contacted for consenting their children’s participation in the study, 51 (65%) accepted. Of these 51 primary school children, 32 (63%) were girls. The daily average minutes of MVPA per day from the accelerometer was 96 (SD 35) and from self-reports was 60 (IQR: 26, 65). Other characteristics of the study participants are shown in Table 1. All raw accelerometer and self-reporting data are available as *Underlying data* ^15^.

**Table 1.** Characteristics and physical activity data for primary school children (N=51).

**Characteristic**	**n (%)**
**Socio demographics** Age (years)	
Mean, SD	(10, 0.8)
9	11 (22)
10	17 (33)
11	23 (45)
Female	32 (63)
School type	
Government	27 (53)
Private	24 (47)
School location	
Moshi urban	33 (65)
Moshi rural	18 (35)
**Accelerometry data** Number of days during entire period for which accelerometer data were available	
3 days	1 (2)
4 days	1 (2)
6 days	1 (2)
7 days	48 (94)
Number of weekdays for which accelerometer data were available	
3 days	2 (4)
4 days	1 (2)
5 days	48 (94)
Number of weekend days for which accelerometer data were available (n = 49) ^§^	
1 day	3 (6)
2 days	46 (94)
Daily average MVPA (minutes)	96 (35) *
**Self-reported physical activity data**	
Number (%) of children reporting:	
Walking to school	29 (57)
Screen time (electronic games, television)	48 (94)
Exercise during school breaks	41 (80)
After school exercises (house chores, games)	44 (86)
Attend physical education sessions (n=47)	36 (77)
Daily average MVPA (minutes)	60 (26, 65) **

MVPA moderate to vigorous physical activity. ^§^Two children were missing valid data for weekend days. *Data are mean (SD). **Data are median (interquartile range), IQR: 25%;75%.

### Correlation between self-reports and accelerometer MVPA

**Figure 2.**
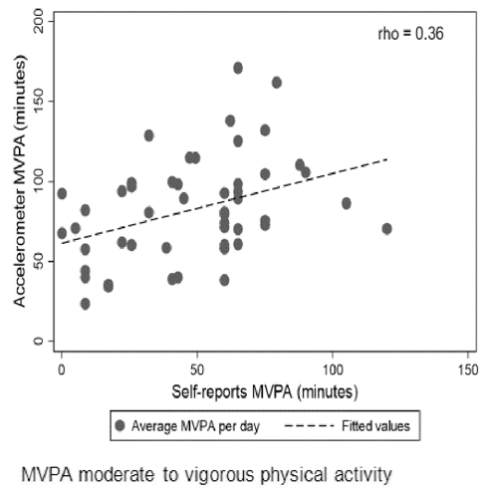
Scatter plot for correlation between average MVPA per day from self-reports and accelerometer MVPA per day, showing a Spearman rho = 0.36.

The correlation between self-reports and accelerometry is presented in Figure 2. Overall, a significant positive correlation was found between accelerometer measured MVPA and self-reported MVPA (rho = 0.36, p=0.009).

**Figure 3.**
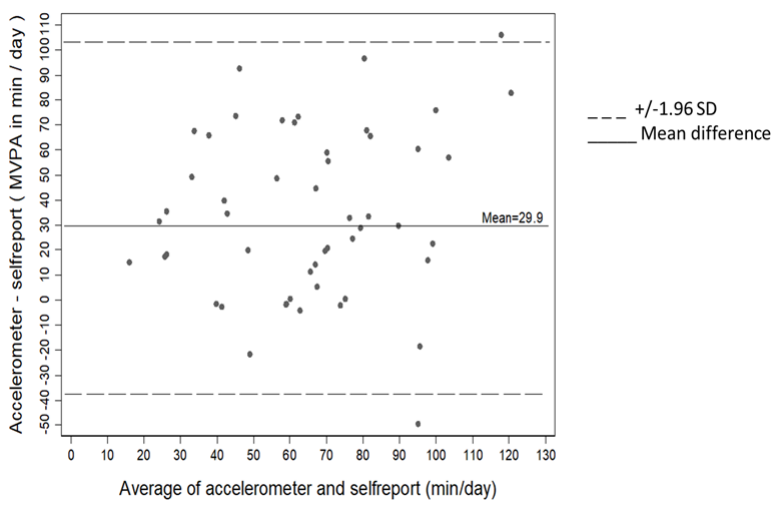
Bland Altman plot of accelerometer and self-reports MVPA, indicating agreements between the two measurements.

The Bland–Altman plot of a difference between self-report and accelerometer MVPA versus a mean of these two methods shows the mean difference of 29.9 minutes of MVPA per day, with the agreement limits ranges from -44 to 104 minutes per day ( Figure 3).

### Variability between self-reports and accelerometer blocks

**Figure 4.**
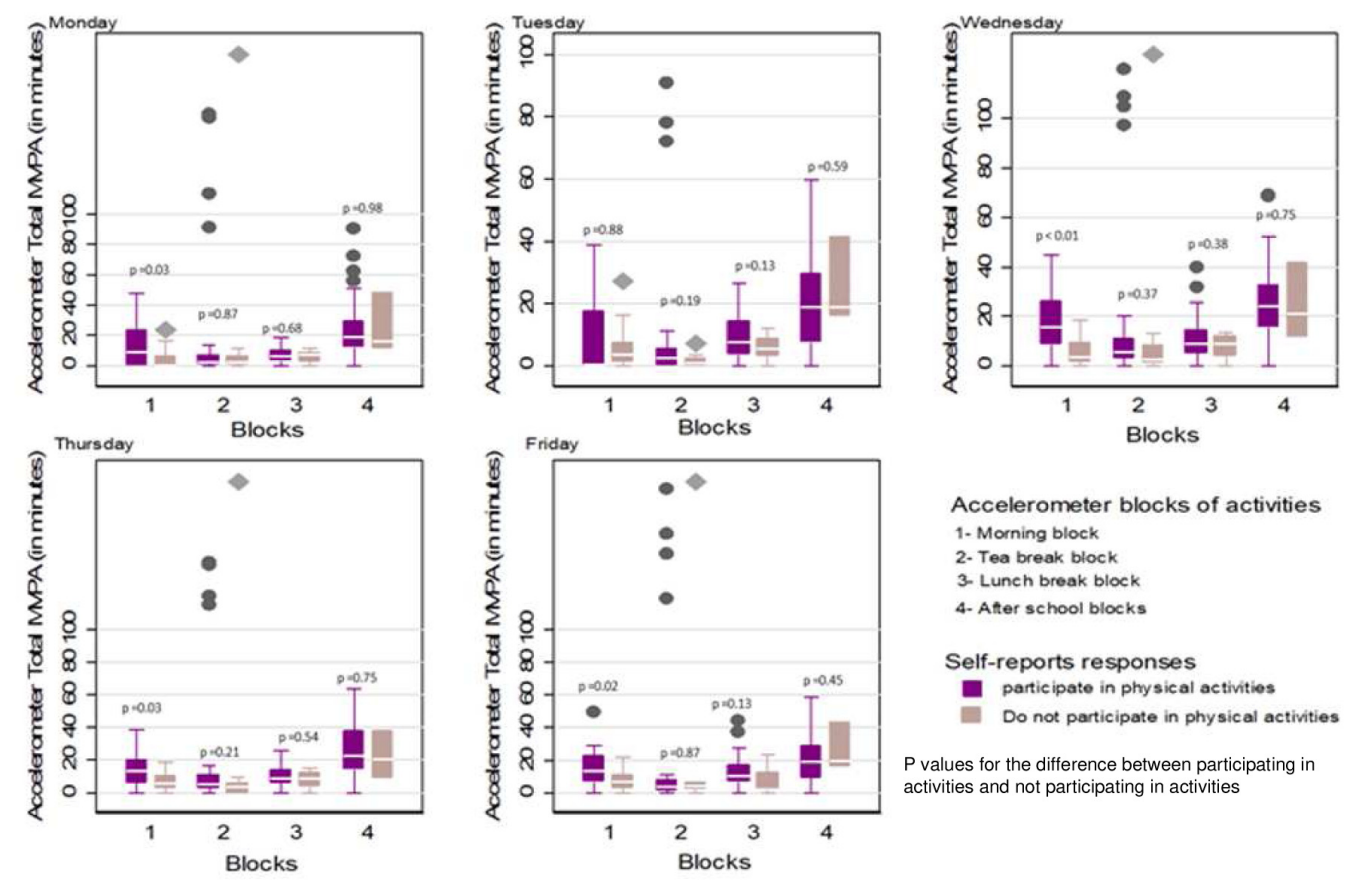
Box and whisker plot presenting accelerometer total MVPA from blocks of activities and self-reports responses across weekdays (school days).

Figure 4 presents the box-and-whisker plots on the comparison of MVPA from time blocks of activities for children who reported participating in activities versus not participating in activities across weekdays (from Monday to Friday). We found a significant difference in median MVPA for children who reported walking to school versus not walking to school block for both days (p < 0.05) except for Tuesdays (p = 0.8). There was no evidence of a difference in median MVPA for other activity blocks across days of the week.

### Mean weekday MVPA

The mean MVPA for the 5 weekdays, Monday – Friday, considered together from self-reports and accelerometer were 261 (SD=179) and 408 (SD=66), respectively ( Table 2). There is evidence of a consistently higher mean MVPA over the 5 weekdays for both accelerometer and self-reports for children who reported walking to school, 480 and 302 (p <0.001) compared to those who don’t walk to school ( Table 2). Similarly, for weekend (Saturday – Sunday) data, the mean accelerometer MVPA was higher than that of self-reported weekend activities ( Table 3).

**Table 2.** Mean moderate to vigorous physical activity (MVPA) over weekdays, Monday – Friday, considered together for self-reports and accelerometer (N=51).

Characteristic	n	Accelerometer		Self-report	
		Mean MVPA ^a^ (95% CI)	p-value	Mean MVPA ^a^ (95% CI)	p-value
Overall ^b^		408 (361- 455)		261 (199 - 323)	
Sex					
Male	19	441 (361-522)	0.3	294 (225 - 362)	0.2
Female	32	388 (328 - 448)		231 (185 - 278)	
Age (years)					
9	11	307 (92 - 523)		248 (164 - 332)	
10	17	457 (296 - 617)	0.06	266 (200 - 331)	0.9
11	23	420 (247 - 592)		242 (184 - 301)	
School type					
Government	27	490 (434 - 547)	<0.001	279 (222 - 335)	0.1
Private	24	315 (255 - 374)		221 (170 - 272)	
School location					
Moshi urban	33	389 (331 - 448)	0.3	253 (199 - 308)	0.9
Moshi rural	18	441 (357 - 526)		248 (202 - 295)	
Walking to school					
Yes	29	480 (419 - 541)	<0.001	302 (254 - 350)	0.002
No	22	313 (258 - 367)		185 (134 - 237)	
Screen time (games, television)					
Yes	47	395 (347 - 444)	0.1	243 (204 - 284)	0.2
No	4	555 (372 -738)		344 (263 - 424)	
Exercise during school breaks					
Yes	41	402 (352 - 453)	0.6	238 (202 - 275)	0.2
No	10	430 (288 - 572)		307 (168 - 445)	
After school exercises					
Yes	44	407 (358 - 456)	0.9	256 (215 - 296)	0.1
No	7	412 (214 - 611)		284 (131 - 438)	
Attend physical education sessions (n=47)					
Yes	36	380 (332 - 428)	0.2	248 (212 - 285)	0.3
No	11	456 (300 - 611)		202 (91 – 313)	

Data are shown in mean MVPA over weekdays (Monday – Friday). ^a ^Mean adjusted for schools as clusters. ^b^Overall mean MVPA for 5 weekdays. CI, confidence interval.

**Table 3.** Mean weekend (Saturday – Sunday) moderate to vigorous physical activity (MVPA) of self-reports and accelerometer (N=49).

Characteristic	n	Accelerometer		n	Self-report	
		Mean MVPA ^a^ (95% CI)	p-value		Mean MVPA ^a^ (95% CI)	p-value
Overall ^b^	49	186 (155 - 218)		24	113 (104 - 121)	
Sex						
Male	17	235 (161 - 309)	0.02	10	108 (90 - 126)	0.37
Female	32	160 (134 -187)		14	116 (107 - 125)	
Age (years)						
9	10	169 (94 - 244)		6	100 (74 -126)	
10	16	226 (164 - 287)	0.21	9	120	0.17
11	23	166 (128 - 204)		9	113 (100 -127)	
School type						
Government	27	231 (190 - 272)	<0.001	13	115 (105 - 125)	0.46
Private	22	131 (93 - 170)		11	109 (93 -125)	
School location						
Moshi urban	32	186 (143 - 228)	0.95	14	107 (92 -122)	0.13
Moshi rural	17	188 (139 - 236)		10	120	
Screen time (electronic games, television)						
Yes	41	183 (149 - 218)	0.66	19	114 (105 - 123)	0.59
No	8	202 (106 - 298)		5	108 (75 - 141)	

MVPA moderate to vigorous physical activity, CI confidence interval. Data are shown in minutes per weekend days (Saturday – Sunday). ^a^Mean adjusted for schools as clusters. ^b^Overall mean MVPA for 5 weekdays. Notes: Two children were missing the weekend's valid data

### Subgroup analysis for associations between child-level variables and total weekday accelerometer MVPA

Table 4 presents the subgroup analysis of the associations between child-level variables and the accelerometer MVPA. In the crude analysis, we found that attending government school (33.6, 95% CI 18.9–48.4) and walking to school (33.4, 95% CI 18.5–48.3) were strongly associated with the total weekday accelerometer-measured MVPA. After accounting for the effect of child-level factors in the multivariable model, only school type remained significantly associated with the total weekday accelerometer-measured MVPA (23.4, 95% CI 4.0–42.8). In contrast, for walking to school, the association was lost. We noted that 32% (ICC) of the variations in accelerometer MVPA were explained by the differences in accelerometer MVPA measurements between one child to another. These differences present a higher variation of MVPA measurements within the same child than the variation between different children.

**Table 4.** Associations between child-level variables and total weekday accelerometer moderate to vigorous physical activity (MVPA) for primary school children (N = 249).

Characteristic	Crude Coefficient (95% CI)	p-value	Adjusted Coefficient (95% CI)	p-value	ICC
**Sex**					
Female	1		1		0.32
Male	12.5 (-5.2-30.1)	0.17	13.2 (-1.0-27.3)	0.07	
**Age (years)**	7.9 (-2.9-18.8)	0.15	5.4 (-4.0-14.8)	0.26	
**School type**					
Private	1		1		
Government	33.6 (18.9-48.4)	<.001	23.4 (4.0-42.8)	0.02	
**School location**					
Moshi urban	1		1		
Moshi rural	11.5 (-6.4-29.3)	0.21	7.7 (-8.8-24.2)	0.36	
**Walking to school**					
No	1		1		
Yes	33.4 (18.5-48.3)	<0.001	13.7 (-6.8-34.3)	0.19	

47 children had a total of 5 days, 4 had either 3 or 4 days) which made a total number of 249 observations (5 days x 47, 3 days x 2, 4 days x 2). ICC, intraclass correlation coefficient; CI, confidence interval.

## Discussion

This study provides evidence regarding self-reports validity to measure physical activity in primary school children in Tanzania. We found a significant positive correlation between self-reports and accelerometry MVPA. Walking to school time block from self-reports corresponded with accelerometer measured activities from this same block.

The significant but moderate positive correlation we observed between self-reports and the accelerometer data can be explained by the inconsistencies of children reporting their actual minutes of MVPA. The level of agreement between these two measurements indicates that the accelerometer measured what self-reports were capturing. The error observed may be due to over-and under-reporting of the actual MVPA. Other validation studies reported similar findings, reflecting the limitations of children’s accuracy to report their actual minutes of MVPA ^8, 20, 21^.

Our findings are consistent with other studies validating physical activity instruments in children. The few studies that evaluated objective measures against children’s self-reported accounts of physical activity in different parts of the world found low to moderate correlations. For instance, a study for tracking physical activity trends in youth aged 10–18 years reported a correlation of 0.27 and 0.34 for boys and girls ^22^. Similarly, other validation studies using accelerometers as a criterion method reported low correlations and documented that most physical activity questionnaires have low to moderate validity ^21– 26^. Together, these data highlight that researchers should interpret self-reported physical activity data with caution due to the limited physical activity assessment validity. However, self-reports are cheaper and easy to capture than objective measurements, and thus researchers can still use them to estimate physical activity levels.

The majority of children reported less time in total MVPA than confirmed by the accelerometer. The most plausible explanation can be due to recall bias. Children are unlikely to remember every minute they participated in physical activity. The accelerometer also captures every physical movement while self-reports followed only a series of questions included in the questionnaire. Differences in activity levels between the self-reports and the accelerometer correspond to what is found in the literature, illustrating the difficulties for children to quantify bouts of activities performed ^7, 23, 27^. Recently, researchers in the Active Healthy Kids Global Alliance, which aims to promote physical activity in children and youth worldwide, pointed out that estimating physical activity prevalence is a worldwide concern. Thus, there is a need for standardized physical assessment surveillance systems in each country ^18^.

A child’s daily routine of walking to school every day, especially for children in government schools, could explain the higher MVPA captured by self-reports and accelerometers. It is possible that this regular activity is easily recalled by school children and well captured by an accelerometer. Furthermore, several activities occur after school daily routine: “after school activities”. For example, running to catch a school bus, playing while waiting for the school bus/ private cars, some children playing on their way home, or participating in several unstructured activities; others participate in household chores. These activities can explain the higher MVPA observed for after school activities. Studies presented in the Global matrix report highlighted that walking to school was a reliable indicator for assessing children's physical activity and youth ^28^.

In the present study, we found that 68% of the daily variation of MVPA was due to day-to-day variability within children, and the effect of a cluster explained 32%. These variations can be explained by the differences in daily activities whereby children may not follow the same activity routine every day. Most government schools’ children walk to school every day while most private schools use private cars / buses to reach school. However, other activities may contribute to the variation in MVPA from day to day, e.g., different activity levels between formal lessons, with some children being active and running around and others just staying in class and being more sedentary. Some studies reported that children's activities depend on their habitual behaviours. Thus, children differ in activity types and levels depending on the time and opportunities to be involved in activities, and supportive environment ^27, 29^. The inclusion of the cluster analysis of the association between child characteristics and accelerometer MVPA could have led to an overestimation of the effect of the child characteristics on MVPA. Further, these variations might contribute to the observed correlations.

This study's strengths include using an objective method, “accelerometers” to validate physical activity questionnaire “self-reports”. To our understanding, this is one of the few studies conducted in resource-restricted countries that aimed to validate the self-reported physical activity questionnaire by applying an objective measurement method. We achieved high compliance with wearing accelerometers; most enrolled children wore them for seven days as instructed. Also, this study explored the effect of the cluster in explaining the variations between individual children’s activities.

In contrast, the limitations of this study need to be acknowledged. There was a high refusal rate from parents with 36% of parents prohibiting their child from participating. The main reason for refusal was fear to use the accelerometers. The denial could have introduced a selection bias to our study as the children who were unable to participate may have been systematically different from those who participated. Further, there are no standard protocols for processing accelerometer data, making interpretation of physical activity data complex.

## Conclusions

This study found a significant positive correlation between self-reports and accelerometers. Self-reports are prone to errors due to recall and social desirability bias which might compromise their validity. Despite these flaws, assessing physical activity using devices is often impossible, especially in low- and middle-income countries due to cost. More research is recommended to develop better self-reported measures with specific physical activities that children can easily recall.

## Data availability

### Underlying data

Figshare: Validation of self-reported physical activity by accelerometry among primary school children in Kilimanjaro, Tanzania: a pilot study. https://doi.org/10.6084/m9.figshare.12763946.v2 ^15^.

This project contains the following underlying data:

- Accelerometry with all days.xlsx. (Raw accelerometry data for each participant.)
- All data_accelerometry_self reports.csv. (Self-reported data for each participant.)

### Extended data

Figshare: Validation of self-reported physical activity by accelerometry among primary school children in Kilimanjaro, Tanzania: a pilot study. https://doi.org/10.6084/m9.figshare.12763946.v2 ^15^.

This project contains the following extended data:

- Bland Altman data.csv. (Data used to generate Bland Altman plots.)
- Box plots data.csv. (Data used to generate box plots.)
- Additional file 1_Self reports questionnaire.docx. (Questionnaire used for self-reporting of activity.)
- Accelerometer time blocks.docx (Description of the different time blocks given for accelerometer data.)

Data are available under the terms of the Creative Commons Zero “No rights reserved” data waiver (CC0 1.0 Public domain dedication).
