# Supplementary material for: Description and comparison of physical activity from self-reports and accelerometry among primary school children in Kilimanjaro, Tanzania: a pilot study
Source: AAS Open Res. 2021 May 17;3:40. Originally published 2020 Aug 21. [Version 4] doi: 10.12688/aasopenres.13118.4 (PMC8132476; doi:10.12688/aasopenres.13118.4)
Supplement: Peer review history

# Peer review: Reviewer response for version 4

**DOI:** 10.21956/aasopenres.14352.r28602

**Published:** 2021-05-18

**Authors:** Ing-Mari Dohrn

**Affiliations:** 1 Department of Neurobiology, Care Sciences and Society (NVS), Karolinska Institutet, Stockholm, Sweden

I thank the authors for their responses and the changes made. I have no further comments.

Is the work clearly and accurately presented and does it cite the current literature?

Partly

If applicable, is the statistical analysis and its interpretation appropriate?

Partly

Are all the source data underlying the results available to ensure full reproducibility?

Yes

Is the study design appropriate and is the work technically sound?

Yes

Are the conclusions drawn adequately supported by the results?

Partly

Are sufficient details of methods and analysis provided to allow replication by others?

Partly

Reviewer Expertise:

NA

I confirm that I have read this submission and believe that I have an appropriate level of expertise to confirm that it is of an acceptable scientific standard.

# Peer review: Reviewer response for version 3

**DOI:** 10.21956/aasopenres.14335.r28558

**Published:** 2021-05-05

**Authors:** Ing-Mari Dohrn

**Affiliations:** 1 Department of Neurobiology, Care Sciences and Society (NVS), Karolinska Institutet, Stockholm, Sweden

Please see my comments in the 2 documents linked below:

1. Full comments
2. Annotated copy of the article

Is the work clearly and accurately presented and does it cite the current literature?

Partly

If applicable, is the statistical analysis and its interpretation appropriate?

Partly

Are all the source data underlying the results available to ensure full reproducibility?

Yes

Is the study design appropriate and is the work technically sound?

Yes

Are the conclusions drawn adequately supported by the results?

Partly

Are sufficient details of methods and analysis provided to allow replication by others?

Partly

Reviewer Expertise:

NA

I confirm that I have read this submission and believe that I have an appropriate level of expertise to confirm that it is of an acceptable scientific standard, however I have significant reservations, as outlined above.

# Response

**Published:** 2021-05-10

**Authors:** Mary Mosha

**Affiliations:** Kilimanjaro Christian Medical University College, Tanzania

Dear Ing-Mari Dohrn,

 Thanks for reviewing this manuscript, as suggested please see below responses.

Comment;

 Table 1 is still confusing: What is the difference between * and **? Which data is mean and sd? It looks like you are only presenting median and IQR.

Response:

 This has been corrected.

 "For accelerometry, sedentary analysis is telling us about the bouts of low activity which gives detailed information about sedentary behavior without considering non-wear time as sedentary time. Total sedentary time includes all times when the device is not worn, sleeping time etc. which might create more confusion. We acknowledge your concern on sedentary analysis, and we hope it is now clear."

Comment:

 I am afraid this is still unclear: What exactly do you mean by: “the daily average of sedentary bouts was 74 (IQR: 48, 118)”?

 What is the average? Is it average number of sedentary bouts? Is it the average total time in sedentary bouts? Is it the average length of a sedentary bout?

 Total sedentary time does not have to include non-weartime. If you cannot sort this out, I suggest that you leave out your sedentary data and only report MVPA.

Response:

 Thanks for your concern, and advice on the sedentary data. We have removed the sedentary information to avoid confusion, as it is also not the main component

# Peer review: Reviewer response for version 3

**DOI:** 10.21956/aasopenres.14335.r28559

**Published:** 2021-04-26

**Authors:** Edvard H. Sagelv

**Affiliations:** 1 School of Sport Sciences, Faculty of Health Sciences, UiT the Arctic University of Norway, Tromsø, Norway

In Figure 4, please write in the figure legend what the p-values represent; i assume they test the difference between activities versus no activities?

 I have no further comments.

Is the work clearly and accurately presented and does it cite the current literature?

Partly

If applicable, is the statistical analysis and its interpretation appropriate?

Partly

Are all the source data underlying the results available to ensure full reproducibility?

Yes

Is the study design appropriate and is the work technically sound?

Yes

Are the conclusions drawn adequately supported by the results?

Yes

Are sufficient details of methods and analysis provided to allow replication by others?

Partly

Reviewer Expertise:

Physical activity measurements, epidemiology, exercise physiology

I confirm that I have read this submission and believe that I have an appropriate level of expertise to confirm that it is of an acceptable scientific standard.

# Response

**Published:** 2021-05-10

**Authors:** Mary Mosha

**Affiliations:** Kilimanjaro Christian Medical University College, Tanzania

Dear Edvard H. Sagelv,

 Thanks for reviewing this manuscript. We have included the information on p values for figure 4 as suggested.

Comment:

 In Figure 4, please write in the figure legend what the p-values represent; i assume they test the difference between activities versus no activities?

Response:

 P values representation added to figure 4.

# Peer review: Reviewer response for version 2

**DOI:** 10.21956/aasopenres.14305.r28292

**Published:** 2021-02-19

**Authors:** Ing-Mari Dohrn

**Affiliations:** 1 Department of Neurobiology, Care Sciences and Society (NVS), Karolinska Institutet, Stockholm, Sweden

I thank the authors for their answers and the changes made, but there are still some issues that must be addressed.

**Abstract:**

 This sentence has not been revised: *“Accelerometer had higher mean MVPA”… *It is not correct grammar to say that “accelerometer had”. You also have to add which outcome it represents.

 I suggest a change to: *The mean total weekday minutes in MVPA from accelerometers were higher than from self-reports, 408 (SD=66) vs 261 (SD=179).*

 Below is my previous comment and the author response. This is still in the abstract, as far as I can see.

- *Maybe leave out since this is not really a validation result, but rather a description of PA* *level: “Children who reported walking to school had higher MVPA for both accelerometer* *and self-reports compared to children who use other means of transport to school, e.g.* *school buses (p < 0.001).”*
**Response:**
  We have removed that section from the abstract as suggested.
- **Methods:**
*Figure 1 is now hard to read. Please only show the vertical axis as this is the cutpoints refer to,* *and clarify the blocks and numbers. In the figure legend, specify if it shows weekday data.*
**Response:**
  We have modified the figure to improve clarity, the figure legend has been improved as suggested. The figure shows one weekday / school day accelerometer output from a single child. More details of blocks descriptions is found in the supplementary file, please see lines 199 – 204.
  The figure is now clearer, but you still show data from all three axes. It would be better to show only data from axis 1, the vertical axis, as this is what the cut-points relate to, or at least state that the cut-points are for axis 1 in the figure legend.
- **Results:**
*Please clarify if your main outcome is mean or median minutes in MVPA per day or total* *MVPA per week? I do not understand how daily MVPA minutes from the accelerometers* *can be median 98 as stated in table 1 and in the results on p 5, when mean weekday MVPA* *minutes are 408 in table 2? It also seems highly unlikely that only 74 minutes per day* *(median) are spent sedentary during a 15 hour day. Are these data in table 1 correct?*
**Response:**
  The MVPA data from table 1 is the average MVPA per day, while for table 2, is the mean MVPA per week. The sedentary time from accelerometer is correct but is now clarified as a daily sedentary bouts of 10 minutes each, please see table 1.
  In Table 1: I think you still show median and IQR for MVPA?
  In Table 2: You still write mean “MVPA for weekdays” and not “mean MVPA per week”.
  Regarding sedentary data: Your write that the data show daily number of 10 min bouts, it that bouts of 10 min each or bouts of at least 10 min each? Median number of bouts is 74, (IQR 48,118) – if the bouts are 10 min each, would that mean that a median min spent sedentary per day is 740 min with IQR: 480-1180 minutes? That equals 12,3 h (IQR 8 h; 19,7 h). Is that realistic? And why do you think that number of bouts are interesting. I cannot see that you are discussing the pattern of sitting or comment on the outcome 10 min bouts. I strongly suggest that you present total number of sedentary minutes? Or at least bouts and total time.
  Regarding figure 4 in the new manuscript. Like the other reviewer I suggest that you remove this figure. It confuses more than it adds, and your interpretation of these results is questionable.

Is the work clearly and accurately presented and does it cite the current literature?

Partly

If applicable, is the statistical analysis and its interpretation appropriate?

Partly

Are all the source data underlying the results available to ensure full reproducibility?

Yes

Is the study design appropriate and is the work technically sound?

Yes

Are the conclusions drawn adequately supported by the results?

Partly

Are sufficient details of methods and analysis provided to allow replication by others?

Partly

Reviewer Expertise:

NA

I confirm that I have read this submission and believe that I have an appropriate level of expertise to confirm that it is of an acceptable scientific standard, however I have significant reservations, as outlined above.

# Response

**Published:** 2021-04-06

**Authors:** Mary Mosha

**Affiliations:** Kilimanjaro Christian Medical University College, Tanzania

Approved with reservations

  

- **Abstract:**
  This sentence has not been revised: *“Accelerometer had higher mean MVPA”… *It is not correct grammar to say that “accelerometer had”. You also have to add which outcome it represents.
  I suggest a change to: *The mean total weekday minutes in MVPA from accelerometers were higher than from self-reports, 408 (SD=66) vs 261 (SD=179).*

**Response:**

 This sentence has been changed as suggested.

  

- *Maybe leave out since this is not really a validation result, but rather a description of PA level: “Children who reported walking to school had higher MVPA for both accelerometer and self-reports compared to children who use other means of transport to school, e.g. school buses (p < 0.001).”*

**Response:**

 We have removed that section from the abstract as suggested.

  

- **Methods:**
Comment:

The figure is now clearer, but you still show data from all three axes. It would be better to show only data from axis 1, the vertical axis, as this is what the cut-points relate to, or at least state that the cut-points are for axis 1 in the figure legend.

**Response:**

 We have modified the figure, and now it shows only one axis (axis 1) which represents physical activity. See lines 148 – 150.

  

- **Results:**
Comment:
  In Table 1: I think you still show median and IQR for MVPA?
  In Table 2: You still write mean “MVPA for weekdays” and not “mean MVPA per week”.
  Regarding sedentary data: Your write that the data show daily number of 10 min bouts, it that bouts of 10 min each or bouts of at least 10 min each? Median number of bouts is 74, (IQR 48,118) – if the bouts are 10 min each, would that mean that a median min spent sedentary per day is 740 min with IQR: 480-1180 minutes? That equals 12,3 h (IQR 8 h; 19,7 h). Is that realistic? And why do you think that number of bouts are interesting. I cannot see that you are discussing the pattern of sitting or comment on the outcome 10 min bouts. I strongly suggest that you present total number of sedentary minutes? Or at least bouts and total time.

**Response 1:**

 Table 1 has been corrected.

 For accelerometry, sedentary analysis is telling us about the bouts of low activity which gives detailed information about sedentary behaviour without considering non-wear time as sedentary time. Total sedentary time includes all times when the device is not worn, sleeping time etc. which might create more confusion. We acknowledge your concern on sedentary analysis, and we hope it is now clear.

**Response 2:**

 Table 2 has been clarified. Results are presented per week. This is now clearly elaborated on the text and table. Therefore Table 2 presents MVPA per week (that is Monday to Friday), while Table 3 presents weekend data.

Comment:

 Regarding figure 4 in the new manuscript. Like the other reviewer I suggest that you remove this figure. It confuses more than it adds, and your interpretation of these results is questionable.

**Response:**

 As per another reviewer, we understand your concern. However, we think this figure is an important part of the paper. The figure reflects the variations of physical activity MVPA which might have contributed to the low to moderate correlations we are observing. To clarify further we used a statistical test to show clearly these differences. See lines 167 – 169, 259 – 261.

# Peer review: Reviewer response for version 2

**DOI:** 10.21956/aasopenres.14305.r28291

**Published:** 2021-02-05

**Authors:** Edvard H. Sagelv

**Affiliations:** 1 School of Sport Sciences, Faculty of Health Sciences, UiT the Arctic University of Norway, Tromsø, Norway

Thank you for the revised version of your manuscript. You have done a good job in revising important aspects of this paper. 

 I have some follow up concerns: 

 I raised this concern in my previous report: 

 " *In page 5 you have a statement in results: “The highest level of activity is seen after school hours and this is consistently captured by both self-reports and accelerometer.” How can I see that from figure 3? The quartiles are all over each other?", *and your response was:

*"* **Response:**

 From figure 3, vertical axis represents the total MVPA captured by accelometer while horizontal presents the blocks of activities. The bars and whisker present the responses from self-reported activities. Thus, from that we can link with the output from accelerometer on the vertical axis. More importantly, the whiskers present the variations of MVPA captured from different blocks of activities between children who participate and not participate in activities"

 Although the median cut off is higher on the y-axis in Figure 4, the variability is high (Q1, Q3, max and min). In my eyes by reading your figure, I cannot say for certain that MVPA is higher after school. For example the IQR seems to be within the IQR of block 3, they may as well overlap to so much extent that they are not different? Thus, whether both accelerometry and self-reported MVPA is higher after school hours compared with in school hours blocks should be tested. I am no statistical expert, so you should consult one. My initial thoughts: An ANOVA might do the work, by setting the time blocks as exposure, however, as these different blocks are the same individuals, I am not sure the ANOVA is correct? Paired t-tests (or the equivalent wilcoxon signed rank test for non-normally distributed data) will work, but here make sure to correct for multiple tests (i.e. per test should the alpha (significance level) be divided; two t-tests, p=0.025 for significant difference, three t-tests, p=0.0125 for significant, and so on. 

 My next comment was also on figure 3. 

*"In page 6, you have a statement in results: “Separately, the accelerometer measured total MVPA during the first block of the day (walking to school) was consistently higher for children who report walking to school compared to those who do not report walking to school or use other means of transport such as private cars or school buses (Figure 3).” Do you have any statistical analysis to back this up? But more importantly, what does this say? That those who produces much acc measured MVPA before school hours also reports higher MVPA in walking to school? Good, but that does not add anything to the validity of the PAQ, the validity of the PAQ is nicely presented in figure 2: correlations and B and A plot."*

 And your response: 

**"Response:**

 Thanks for the comment, we do not have any statistical test for this, rather the median and quartiles. This figure tries to confirm or compare if at all those children who reported yes/no, they participate in physical activity is well captured by accelerometer."

 Okay, I accept your intent, however, your interpretation might be wrong, as you do not test this. I strongly urge to either 1) perform statistical analyses testing whether there are in reality differences among those who report yes and no, and between blocks. The median and IQR are in my view overlapping many places. Thus, your interpretation may be overrated by used looking at the figure. A second way of dealing with this is to just omit figure 3. Your paper is nice as it is without that figure. I would have done the last thing here, just omitting figure 3. A last option is to present it and not comment on it, i.e. whether one block is higher than the other in terms of MVPA (it is here I do not follow and I fear your interpretation by just looking at the figure might be mistaken).

 My third comment here also is about the same theme: 

 " *Table 2: ok, good. Here comes the states about MVPA before school etc. then, I do not see what figure 3 and the box and whisker add, and the whole variability/bias in methods add.*

**"Response**:

 Thanks for the suggestion, we included the box and whisker plots to understand if accelerometer time blocks output (MVPA) and self-reports responses are matching. E.g. We were expecting to see the higher MVPA from children who said yes, they walk to school than those who don’t."

 So whether acc and self-report matches is presented nicely in the bland and altman plot. Table 2 present the data and test for whether walking to school gives higher acc MVPA, and whether after school exercise gives more MVPA (which it did not). so, you do not need figure 3, i think many readers will face my problem in understanding what it is and what it represents. 

*Table 2: ok, good. Here comes the states about MVPA before school etc. then, I do not see what figure 3 and the box and whisker add, and the whole variability/bias in methods add.*

 Here is another response you had to my comment on low/moderate validity:

**"Response:**

 We have included the word criterion as suggested. Low to moderate validity depends on the nature of the study, that is there is no specific cut off points.  For most studies of these nature validity was described as the strength of the correlations and ranked as small (>0.1), moderate (>0.3) and strong (>0.5)"

 Exactly: therefore, you should have presented the definition for low and moderate validity in parentheses. e.g. low validity (pearson´s r<0.01) in your text.

 Finally, I still find some typos all over the text, however, these typos are not influencing the message of the sentences, thus, a reader will clearly understand what is meant. However, if possible, consider reading carefully through your text to remove typos. 

 All other comments are appropriately addressed and answered, and I have no further comments.

 I would like to again congratulate the authors on a nice study, a good job and a good presentation your data from a part of the world where this is much needed. Well done.

Is the work clearly and accurately presented and does it cite the current literature?

Partly

If applicable, is the statistical analysis and its interpretation appropriate?

Partly

Are all the source data underlying the results available to ensure full reproducibility?

Yes

Is the study design appropriate and is the work technically sound?

Yes

Are the conclusions drawn adequately supported by the results?

Yes

Are sufficient details of methods and analysis provided to allow replication by others?

Partly

Reviewer Expertise:

Physical activity measurements, epidemiology, exercise physiology

I confirm that I have read this submission and believe that I have an appropriate level of expertise to confirm that it is of an acceptable scientific standard.

# Response

**Published:** 2021-04-06

**Authors:** Mary Mosha

**Affiliations:** Kilimanjaro Christian Medical University College, Tanzania

Approved

 Thank you for the revised version of your manuscript. You have done a good job in revising important aspects of this paper. 

 I have some follow up concerns: 

 Although the median cut off is higher on the y-axis in Figure 4, the variability is high (Q1, Q3, max and min). In my eyes by reading your figure, I cannot say for certain that MVPA is higher after school. For example, the IQR seems to be within the IQR of block 3, they may as well overlap to so much extent that they are not different? Thus, whether both accelerometry and self-reported MVPA is higher after school hours compared with in school hours blocks should be tested. I am no statistical expert, so you should consult one. My initial thoughts: An ANOVA might do the work, by setting the time blocks as exposure, however, as these different blocks are the same individuals, I am not sure the ANOVA is correct? Paired t-tests (or the equivalent wilcoxon signed rank test for non-normally distributed data) will work, but here make sure to correct for multiple tests (i.e. per test should the alpha (significance level) be divided; two t-tests, p=0.025 for significant difference, three t-tests, p=0.0125 for significant, and so on.                                        

**Response:**

 We used Mann Whitney U test to examine the differences between medians of activities measured by accelerometer versus those activities measured by self-reports. See lines 167 – 169, 259 – 261.

Comment:

 Okay, I accept your intent, however, your interpretation might be wrong, as you do not test this. I strongly urge to either 1) perform statistical analyses testing whether there are in reality differences among those who report yes and no, and between blocks. The median and IQR are in my view overlapping many places. Thus, your interpretation may be overrated by used looking at the figure. A second way of dealing with this is to just omit figure 4. Your paper is nice as it is without that figure. I would have done the last thing here, just omitting figure 4. A last option is to present it and not comment on it, i.e. whether one block is higher than the other in terms of MVPA (it is here I do not follow and I fear your interpretation by just looking at the figure might be mistaken).

**Response:**

 Thanks for your concern on figure 4 (The box and whisker plot).  We would like to acknowledge your important comments on this figure. We still think this figure adds an important information on this paper, as what we are learning from this figure might have contributed to the low to moderate correlations found between self-reports and accelerometers. However, we have now performed a statistical test to clearly understand the differences in medians between different blocks of activities. See lines 167 – 169, 259 – 261.

Comment:

 Exactly: therefore, you should have presented the definition for low and moderate validity in parentheses. e.g. low validity (Pearson's r<0.01) in your text.

**Response:**

 This has been considered and mentioned in the text. See lines 160 – 161.

Comment

 Finally, I still find some typos all over the text, however, these typos are not influencing the message of the sentences, thus, a reader will clearly understand what is meant. However, if possible, consider reading carefully through your text to remove typos. 

**Response:**

 We have corrected typos.

# Peer review: Reviewer response for version 1

**DOI:** 10.21956/aasopenres.14218.r28147

**Published:** 2020-11-26

**Authors:** Edvard H. Sagelv

**Affiliations:** 1 School of Sport Sciences, Faculty of Health Sciences, UiT the Arctic University of Norway, Tromsø, Norway

**General comment:**

 Thank you for the opportunity to review this paper. This paper assessed the criterion validity of self-report in children in Tanzania. The study is well conducted and the results support the conclusion. The language and structure of the manuscript is good, and the reading flows nicely. Overall, this manuscript holds high quality in all parts.

 My major concern is Figure 3. I do not understand it, or the time blocks to assess for the bias. Could you please elaborate and/or at least make it easier to understand?

 Below follow minor concerns. My biggest comment here is the long conclusion, that in my view could benefit from being shorter.

**Abstract: **

- Results: “Accelerometer had higher MVPA compared to self-reports.” Please report some statistical output here. A t-test results f.ex.
- “Conclusions: This study found the moderate positive correlation between self-reports and accelerometers.” – please replace “the moderate..” with “a moderate..”.

**Intro:**

- Statement: “Accelerometers provide valid estimates of activity levels by capturing movement in real time and having low technical error of measurement” – You need a reference for this. However, whether the estimates from accelerometry is valid can be debated. I suggest to state: *“more valid than self-report when evaluated against doubly labelled water estimated energy expenditure”* or something similar.
- Statement: “The validity of self-reports for assessing physical activity in primary school children have not been fully explored in our setting. It is unclear if children from Tanzania can accurately report their physical activity, or estimate minutes spent in physical activity.” – just omit the last sentence here and replace “our setting” with “Tanzania” in the first sentence.

**Methods: **

- Shapiro-wilk: so how did your normality distribution turn out? I assume acc MVPA are positively skewed due to the acc intensity cut-offs? Please report the results from your normality tests. F.ex. you use spearman as I assume MPVA is not normally distributed?#
- You mention moderate correlation in results, but I do not see any reference to strength of correlation cut-offs under statistical analysis?
- Sub-group analysis: in my view, this is far from your aim and adds little to any validity study? You may consider to place all these things in supplementary? Or further.
- Bias/time blocks/Figure 3: the box and whisker plots. I do not follow this figure, nor do I follow your chosen blocks. Those in purple reported in the PAQ to perform MVPA in that specific time period (morning, tea, lunch, after school), while those in pink reported not to? If so, you lined the median and quartiles, and you have no outliers? So, what is that used for? This figure shows accelerometry detected MVPA in those reporting no MVPA to, so I do not follow. Could you please make this easier for a reader to follow? So, on nr 4: after school, the no PA reporters are all over the acc measured MVPA?

**Results:**

- In page 5 you have a statement in results: *“The highest level of activity is seen after school hours and this is consistently captured by both self-reports and accelerometer.”* How can I see that from figure 3? The quartiles are all over each other?
- In page 6, you have a statement in results: *“Separately, the accelerometer measured total MVPA during the first block of the day (walking to school) was consistently higher for children who report walking to school compared to those who do not report walking to school or use other means of transport such as private cars or school buses (Figure 3).”* Do you have any statistical analysis to back this up? But more importantly, what does this say? That those who produces much acc measured MVPA before school hours also reports higher MVPA in walking to school? Good, but that does not add anything to the validity of the PAQ, the validity of the PAQ is nicely presented in figure 2: correlations and B and A plot.
- Table 2: ok, good. Here comes the states about MVPA before school etc. then, I do not see what figure 3 and the box and whisker add, and the whole variability/bias in methods add.

**Discussion**

- Para 2: typo: “a moderate correlation observed”, should be “a moderate correlation was observed.”
- Para 3: typo: “a few studies”, should be “few studies”. This first sentence in this para needs rephrasing: my suggestion: *“Our findings are consistent with other studies validating self-reported physical activity instruments in children”* – you cite quite many studies further into the para, so few studies is perhaps an overstatement?
- Para 3: *“Similarly, other validation studies using accelerometers as a reference method, reported low correlations and documented that most physical activity questionnaires have low to moderate validity19,21–27”* replace “acc as reference” to “acc as the criterion”, as you and these studies report on the acc as the criterion method. Further, what is low to moderate validity?
- Para 3: “…and speak to the need for using both methods i.e objective measures and self-reports where possible.”” Sure, but more importantly, as you mention in the intro, perhaps not all can afford ACCs, so in my view, the most important take home is: “…this highlight that researchers should interpret self-reported PA data with caution due to the imprecise assessment of PA.” but still, they are not way off, indicating that it is better than no assessment.
- Para 4: first sentence, typo: “…confirmed by acc”. Should be “… confirmed by the acc”.
- Para 4: alternatively, the most plausible explanation is that recall bias influences this. How can a 10-year old precicely remember all MVPA minutes? I am not 10 years old, but still, I cannot recall every MVPA minute I did.
- Para 4: here are recall. I simply suggest to restructure this para. Make recall come first.
- Para 5: I refer the authors to my statement in methods: this is outside the aims, consider supplementary or omit.
- Para 6: there is no gold standard for assessing PA (Hills AP, Mokhtar N, Byrne NM.. *Front Nutr*
- 2014;1:5; Westerterp KR. *Eur J Appl Physiol *2009;105:823–8). This is no strength of the study. Please omit. The high compliance is a strength.
- Limitations: the refuse rate is a limitation. The nature of self-report PA, i.e. recall bias, is not a limitation OF this study. This study aimed to assess the validity of self-reported PA.
- A limitation can be the no standard of acc protocol for processing the data?

**Conclusion:**

- Quite long. I suggest to cut it after: *“* *This study found the moderate positive correlation between self-reports and accelerometers. Self-reports are prone to errors due to recall bias which might interfere their validity. Despite these flaws, assessing physical activity using devices is often not possible, especially in low- and middle-income countries due to cost.”* The other things you mention: “Secondly…”, that is suitable for the discussion.

Is the work clearly and accurately presented and does it cite the current literature?

Partly

If applicable, is the statistical analysis and its interpretation appropriate?

Partly

Are all the source data underlying the results available to ensure full reproducibility?

Yes

Is the study design appropriate and is the work technically sound?

Yes

Are the conclusions drawn adequately supported by the results?

Yes

Are sufficient details of methods and analysis provided to allow replication by others?

Partly

Reviewer Expertise:

Physical activity measurements, epidemiology, exercise physiology

I confirm that I have read this submission and believe that I have an appropriate level of expertise to confirm that it is of an acceptable scientific standard, however I have significant reservations, as outlined above.

# Response

**Published:** 2021-01-05

**Authors:** Mary Mosha

**Affiliations:** Kilimanjaro Christian Medical University College, Tanzania

***General comment:***

*Thank you for the opportunity to review this paper. This paper assessed the criterion validity of self-report in children in Tanzania. The study is well conducted and the results support the conclusion. The language and structure of the manuscript is good, and the reading flows nicely. Overall, this manuscript holds high quality in all parts.*

**

*My major concern is Figure 3. I do not understand it, or the time blocks to assess for the bias. Could you please elaborate and/or at least make it easier to understand?*

**Response:**

 Thanks for this concern, figure 3 is now elaborated to make it easier to understand

 Below follow minor concerns. My biggest comment here is the long conclusion, that in my view could benefit from being shorter.

***Abstract: ***

- *Results: “Accelerometer had higher MVPA compared to self-reports.” Please report some statistical output here. A t-test results f.ex.*

* Response*

*We have added the mean MVPA differences and standard deviation.*

- *“Conclusions: This study found the moderate positive correlation between self-reports and accelerometers.” – please replace “the moderate..” with “a moderate..”.*

**Response:**

 We have corrected the sentence as suggested, “the moderate” is replaced with “a positive significant”.

***Intro:***

- *Statement: “Accelerometers provide valid estimates of activity levels by capturing movement in real time and having low technical error of measurement” – You need a reference for this. However, whether the estimates from accelerometry is valid can be debated. I suggest to state: “more valid than self-report when evaluated against doubly labelled water estimated energy expenditure” or something similar.*

**Response:**

 We have worked on the sentence, and it now reads as “Accelerometers are more valid than self-reports in estimating physical activity, however some studies suggest doubly labeled water method as a gold standard to measure energy expenditure related physical activity”

  

- *Statement: “The validity of self-reports for assessing physical activity in primary school children have not been fully explored in our setting. It is unclear if children from Tanzania can accurately report their physical activity, or estimate minutes spent in physical activity.” – just omit the last sentence here and replace “our setting” with “Tanzania” in the first sentence.*

**Response:**

 We have added the word Tanzania, and the last sentence is omitted it now reads “It is unclear if children from Tanzania can accurately report their physical activity, or estimate minutes spent in physical activity. Please see line 79.

***Methods: ***

- *Shapiro-wilk: so how did your normality distribution turn out? I assume acc MVPA are positively skewed due to the acc intensity cut-offs? Please report the results from your normality tests. F.ex. you use spearman as I assume MPVA is not normally distributed?#*

**Response:**

 We have presented the results from Shapiro Wilk test.

- *You mention moderate correlation in results, but I do not see any reference to strength of correlation cut-offs under statistical analysis?*

**Response:**

 We would like to acknowledge your observation on the strength of correlation. The decision of the cutoffs was informed by the previous studies as indicated under discussion section references 19 - 25.

- *Sub-group analysis: in my view, this is far from your aim and adds little to any validity study? You may consider to place all these things in supplementary? Or further.*

**Response:**

 Thank you for the important concern, we thought it is important to understand the effect of the cluster on MVPA. We therefore performed the subgroup analysis to explore the associations (sex, age, school type and school location), considering clustering effect (child) in order to understand the effect of cluster on MVPA, which could explain the amount of variability in child MVPA

*Bias/time blocks/Figure 3: the box and whisker plots. I do not follow this figure, nor do I follow your chosen blocks. Those in purple reported in the PAQ to perform MVPA in that specific time period (morning, tea, lunch, after school), while those in pink reported not to? If so, you lined the median and quartiles, and you have no outliers? So, what is that used for? This figure shows accelerometry detected MVPA in those reporting no MVPA to, so I do not follow. Could you please make this easier for a reader to follow? So, on nr 4: after school, the no PA reporters are all over the acc measured MVPA?*

**Response:**

 This has been elaborated, and included the outliers as suggested. 

***Results:***

- *In page 5 you have a statement in results: “The highest level of activity is seen after school hours and this is consistently captured by both self-reports and accelerometer.” How can I see that from figure 3? The quartiles are all over each other?*

**Response:**

 From figure 3, vertical axis represents the total MVPA captured by accelometer while horizontal presents the blocks of activities. The bars and whisker present the responses from self-reported activities. Thus, from that we can link with the output from accelerometer on the vertical axis. More importantly, the whiskers present the variations of MVPA captured from different blocks of activities between children who participate and not participate in activities

- *In page 6, you have a statement in results: “Separately, the accelerometer measured total MVPA during the first block of the day (walking to school) was consistently higher for children who report walking to school compared to those who do not report walking to school or use other means of transport such as private cars or school buses (Figure 3).” Do you have any statistical analysis to back this up? But more importantly, what does this say? That those who produces much acc measured MVPA before school hours also reports higher MVPA in walking to school? Good, but that does not add anything to the validity of the PAQ, the validity of the PAQ is nicely presented in figure 2: correlations and B and A plot.*

**Responses:**

 Thanks for the comment, we do not have any statistical test for this, rather the median and quartiles. This figure tries to confirm or compare if at all those children who reported yes/no, they participate in physical activity is well captured by accelerometer.

*Table 2: ok, good. Here comes the states about MVPA before school etc. then, I do not see what figure 3 and the box and whisker add, and the whole variability/bias in methods add.*

**Response**:

 Thanks for the suggestion, we included the box and whisker plots to understand if   accelerometer time blocks output (MVPA) and self-reports responses are matching. Eg. We were expecting to see the higher MVPA from children who said yes, they walk to school than those who don’t.

***Discussion***

- *Para 2: typo: “a moderate correlation observed”, should be “a moderate correlation was observed.”*

**Response:**

 We have corrected the paragraph and the word ‘’was” added.

  

- *Para 3: typo: “a few studies”, should be “few studies”. This first sentence in this para needs rephrasing: my suggestion: “Our findings are consistent with other studies validating self-reported physical activity instruments in children” – you cite quite many studies further into the para, so few studies is perhaps an overstatement?*

**Response:**

 We have corrected the typo. We cited several studies to show the range of correlations from different studies of the same nature, and how were they reported.

  

- *Para 3: “Similarly, other validation studies using accelerometers as a reference method, reported low correlations and documented that most physical activity questionnaires have low to moderate validity19,21–27” replace “acc as reference” to “acc as the criterion”, as you and these studies report on the acc as the criterion method. Further, what is low to moderate validity?*

**Response:**

 We have included the word criterion as suggested. Low to moderate validity depends on the nature of the study, that is there is no specific cut off points.  For most studies of these nature validity was described as the strength of the correlations and ranked as small (>0.1), moderate (>0.3) and strong (>0.5)

  

- *Para 3: “…and speak to the need for using both methods i.e objective measures and self-reports where possible.”” Sure, but more importantly, as you mention in the intro, perhaps not all can afford ACCs, so in my view, the most important take home is: “…this highlight that researchers should interpret self-reported PA data with caution due to the imprecise assessment of PA.” but still, they are not way off, indicating that it is better than no assessment.*

**Response:**

 Thanks for this comment, we have added some more details as suggested and it now reads “these data highlight that researchers should interpret self-reported physical activity data with caution due to the limited validity in assessment of PA. However, self-reports are cheaper and easy to administer than objective measurements, and thus they can still be used to estimate physical activity levels”.

- *Para 4: first sentence, typo: “…confirmed by acc”. Should be “… confirmed by the acc”.*

**Response:**

 We have corrected they typo as suggested, please see line 335. 

- *Para 4: alternatively, the most plausible explanation is that recall bias influences this. How can a 10-year old precicely remember all MVPA minutes? I am not 10 years old, but still, I cannot recall every MVPA minute I did.*
- *Para 4: here are recall. I simply suggest to restructure this para. Make recall come first.*

**Response:**

 We have restructured paragraph 4 as suggested.

  

- *Para 5: I refer the authors to my statement in methods: this is outside the aims, consider supplementary or omit.*

**Response:**

 Thanks for this concern, we have modified the title to include this information as it was important to understand the relevance of reporting physical activity from children as compared with accelerometry.

  

- *Para 6: there is no gold standard for assessing PA (Hills AP, Mokhtar N, Byrne NM.. Front Nutr*
- *2014;1:5; Westerterp KR. Eur J Appl Physiol 2009;105:823–8). This is no strength of the study. Please omit. The high compliance is a strength.*

**Response:**

 We have reviewed this paper and changed the first part of the paragraph as suggested. 

- *Limitations: the refuse rate is a limitation. The nature of self-report PA, i.e. recall bias, is not a limitation OF this study. This study aimed to assess the validity of self-reported PA.*
- *A limitation can be the no standard of acc protocol for processing the data?*

**Response:**

 We have included more details on this section.

***Conclusion:***

- Quite long. I suggest to cut it after: *“This study found the moderate positive correlation between self-reports and accelerometers. Self-reports are prone to errors due to recall bias which might interfere their validity. Despite these flaws, assessing physical activity using devices is often not possible, especially in low- and middle-income countries due to cost.”* The other things you mention: “Secondly…”, that is suitable for the discussion.

**Response:**

 We have reduced the conclusion as suggested.

# Peer review: Reviewer response for version 1

**DOI:** 10.21956/aasopenres.14218.r27869

**Published:** 2020-09-29

**Authors:** Ing-Mari Dohrn

**Affiliations:** 1 Department of Neurobiology, Care Sciences and Society (NVS), Karolinska Institutet, Stockholm, Sweden

The findings of the presented manuscript add value to the existing knowledge in this field of research. However, there are some minor and major aspects that need to be addressed by the authors before approval.  

**Title:**

- The authors present quite a lot of data that is more of a description of PA level and pattern in the examined group in addition to the validation data, and may want to change the title to include that, or exclude some of the presented results.

**Abstract:**

- Please omit “using a simple random sampling technique” as this implies that is was not a convenience sample.
- Change to “A moderate, positive correlation was found between self-reports and accelerometer for weekday MVPA.. “ and  “Accelerometer data showed more time in MVPA..”
- Maybe leave out since this is not really a validation result, but rather a description of PA level: “Children who reported walking to school had higher MVPA for both accelerometer and self-reports compared to children who use other means of transport to school, e.g. school buses (p < 0.001).”

**Introduction:**

- Please add: “In Tanzania, 82.1% of school-going children are not meeting the recommended physical activity levels according to self-reports.”
- The sentence: “Objective measures have been widely used in high-income… can be replaced by this sentence further down “Over the years, accelerometers have been used increasingly in high-income countries for assessment of physical activity in children ^11,12^. Methods include…”
- Please change "self-reports" in the aim to a modified questionnaire.

 

**Methods:** 

- I suggest the following changes:
  "The World Health Organization (WHO) recommends an average of at least 60 minutes per day of moderate to vigorous physical activity (MVPA) for 5–17-year-olds…" and “Our main study variable was minutes per day spent in MVPA which was obtained from self-reports and accelerometry.” I guess it is not minutes per week?
- Please include the final set of questions that you used, in this section. The reader should be able to understand how the study was performed without having to go an appendix to get information.
- Did you ask the children to note any removals of the accelerometer during the day? How did you treat non-wear time during the day? Did you have a minimum hours of wear time for a valid day?
- Figure 1 is now hard to read. Please only show the vertical axis as this is the cutpoints refer to, and clarify the blocks and numbers. In the figure legend, specify if it shows weekday data.

 

**Results:**

- Please clarify if your main outcome is mean or median minutes in MVPA per day or total MVPA per week? I do not understand how daily MVPA minutes from the accelerometers can be median 98 as stated in table 1 and in the results on p 5, when mean weekday MVPA minutes are 408 in table 2? It also seems highly unlikely that only 74 minutes per day (median) are spent sedentary during a 15 hour day. Are these data in table 1 correct?
- Figure 2: A) should the value for r be 0.36? B) It is unclear if the outer dotted lines are ± 1.96 SD? It is not necessary to report CI for the mean difference (inner dotted lines), it only makes the graph hard to read.
- Figure 3: It is unclear how the pink and purple boxplots represent children participating in physical activity or not. What was this classification based on? This is not explained in the figure legend.
- The ICC calculations and the results presented in table 3 and 4 are only relevant if they are used for validation of the questions. For example, is the self-report questions more valid for boys or girls or children in one type of school. Please revise.
- In the discussion you state that “Walking to school and afterschool activity blocks from self-reports corresponds with accelerometer measured activities from these blocks.” Please present clearly these results and how this was analyzed.
- Please comment on why you did not use weekend data in the validation or data on sedentary time. I think this would strengthen the results.

Is the work clearly and accurately presented and does it cite the current literature?

Partly

If applicable, is the statistical analysis and its interpretation appropriate?

Partly

Are all the source data underlying the results available to ensure full reproducibility?

Yes

Is the study design appropriate and is the work technically sound?

Yes

Are the conclusions drawn adequately supported by the results?

Partly

Are sufficient details of methods and analysis provided to allow replication by others?

Partly

Reviewer Expertise:

My areas of expertise are physical activity and sedentary behavior and associations with health, and objective assessment methods for physical activity and sedentary behavior, such as accelerometry.

I confirm that I have read this submission and believe that I have an appropriate level of expertise to state that I do not consider it to be of an acceptable scientific standard, for reasons outlined above.

# Response

**Published:** 2021-01-05

**Authors:** Mary Mosha

**Affiliations:** Kilimanjaro Christian Medical University College, Tanzania

*The findings of the presented manuscript add value to the existing knowledge in this field of research. However, there are some minor and major aspects that need to be addressed by the authors before approval.*

**

***Title:***

- *The authors present quite a lot of data that is more of a description of PA level and pattern in the examined group in addition to the validation data and may want to change the title to include that or exclude some of the presented results.*

**Response:**

 We have altered the title to reflect reviewers’ comments. Please see line 1

***Abstract:***

- *Please omit “using a simple random sampling technique” as this implies that is was not a convenience sample.*

**Response:**

 We have revised this section and clarify, it is now written as “Four primary schools conveniently selected in Moshi municipal and Moshi rural districts, Kilimanjaro, Tanzania.  A total of 51 primary school children aged 9 – 11 years were randomly selected from the 4 schools”. Please see lines 22 - 24

  

- *Change to “A moderate, positive correlation was found between self-reports and accelerometer for weekday MVPA.. “ and  “Accelerometer data showed more time in MVPA..”*

**Response:**

 We have changed the sentence as suggested, please see line 33

  

- *Maybe leave out since this is not really a validation result, but rather a description of PA level: “Children who reported walking to school had higher MVPA for both accelerometer and self-reports compared to children who use other means of transport to school, e.g. school buses (p < 0.001).”*

**Response:**

 We have removed that section from the abstract as suggested.

***Introduction:***

- *Please add: “In Tanzania, 82.1% of school-going children are not meeting the recommended physical activity levels according to self-reports.”*

**Response:**

 We have added the sentence as suggested, please see line 55

  

- *The sentence: “Objective measures have been widely used in high-income… can be replaced by this sentence further down “Over the years, accelerometers have been used increasingly in high-income countries for assessment of physical activity in children ^11,12^. Methods include…”*

**Response:**

 We have replaced the sentences as suggested, see lines 66 - 76

  

- *Please change "self-reports" in the aim to a modified questionnaire.*

**Response:**

 We have changed this sentence, please see line 80 

***Methods:** *

- *I suggest the following changes:*
* "The World Health Organization (WHO) recommends an average of at least 60 minutes per day of moderate to vigorous physical activity (MVPA) for 5–17-year-olds…" and “Our main study variable was minutes per day spent in MVPA which was obtained from self-reports and accelerometry.” I guess it is not minutes per week?*

**Response:**

 We have made changes so that it is now clear, please see lines 98 – 99.

- *Please include the final set of questions that you used, in this section. The reader should be able to understand how the study was performed without having to go an appendix to get information.*

**Response:**

 We have now included the final set of questions used as suggested, please see lines 129 - 139

- *Did you ask the children to note any removals of the accelerometer during the day? How did you treat non-wear time during the day? Did you have a minimum hours of wear time for a valid day?*

**Response:**

 Thank you for this question. Children were told to note the time when they remove the accelerometers, but it didn’t work out. However, with the Actigraph software you can determine the non-wear time, and we defined non wear time during analysis. This section is now elaborated, please see lines 193 - 196

  

- *Figure 1 is now hard to read. Please only show the vertical axis as this is the cutpoints refer to, and clarify the blocks and numbers. In the figure legend, specify if it shows weekday data.*

**Response:**

 We have modified the figure to improve clarity, the figure legend has been improved as suggested. The figure shows one weekday / school day accelerometer output from a single child. More details of blocks descriptions is found in the supplementary file, please see lines 199 - 204

***Results:***

- *Please clarify if your main outcome is mean or median minutes in MVPA per day or total MVPA per week? I do not understand how daily MVPA minutes from the accelerometers can be median 98 as stated in table 1 and in the results on p 5, when mean weekday MVPA minutes are 408 in table 2? It also seems highly unlikely that only 74 minutes per day (median) are spent sedentary during a 15 hour day. Are these data in table 1 correct?*

**Response:**

 The MVPA data from table 1 is the average MVPA per day, while for table 2, is the mean MVPA per week. The sedentary time from accelerometer is correct but is now clarified as a daily sedentary bouts of 10 minutes each, please see table 1

  

- *Figure 2: A) should the value for r be 0.36? B) It is unclear if the outer dotted lines are ± 1.96 SD? It is not necessary to report CI for the mean difference (inner dotted lines), it only makes the graph hard to read.*

**Response:**

 We have corrected the figure, and CI removed from the figure, please see figure 2

- *Figure 3: It is unclear how the pink and purple boxplots represent children participating in physical activity or not. What was this classification based on? This is not explained in the figure legend.*

**Response:**

 We have improved the figure legend, and elaborate more on the document that this figure compares the total MVPA from accelerometer output with children who either said yes they participate in physical activity or no they don’t . Please see lines 219 – 222, and figure 3

  

- *The ICC calculations and the results presented in table 3 and 4 are only relevant if they are used for validation of the questions. For example, is the self-report questions more valid for boys or girls or children in one type of school. Please revise.*

**Response:**

 We performed the analysis to explore the associations between weekdays accelerometer MVPA and different child level variables, since child was having repeated measurements from accelerometer i.e. Monday to Friday we regarded child as a cluster. This helped to understand the effect of a child as cluster on accelerometer MVPA. i.e understanding how much of the variations in MVPA is contributed by child.

  

- *In the discussion you state that “Walking to school and afterschool activity blocks from self-reports corresponds with accelerometer measured activities from these blocks.” Please present clearly these results and how this was analyzed.*

**Response:**

 Box and whisker plots were plotted to present these findings. The horizontal axis informs us about the total MVPA captured by accelerometry from the children who said they participate in certain activities versus those who they don’t participate (from self-reports). Therefore for walking to school and afterschool activity blocks, we see high levels of total MVPA which indicates that participation in these activities was well captured by accelerometer. 

*Please comment on why you did not use weekend data in the validation or data on sedentary time. I think this would strengthen the results.*

**Response:**

 Thanks for sharing this concern, we targeted school days as we expected that during school days children are participating in a set of activities which can easily be remembered than weekends in which there are several unstructured activities in which we could not capture by a questionnaire. However, some of the weekend data are presented in table 3. This is elaborated in lines  210 - 213
